# Identification of White Matter Networks Engaged in Object (Face) Recognition Showing Differential Responses to Modulated Stimulus Strength

**DOI:** 10.1093/texcom/tgaa067

**Published:** 2020-09-18

**Authors:** Muwei Li, Zhaohua Ding, John C Gore

**Affiliations:** Vanderbilt University Institute of Imaging Science, Vanderbilt University, Nashville, TN 37232-2310, USA; Department of Radiology and Radiological Sciences, Vanderbilt University Medical Center, Nashville, TN 37232, USA; Vanderbilt University Institute of Imaging Science, Vanderbilt University, Nashville, TN 37232-2310, USA; Department of Biomedical Engineering, Vanderbilt University, Nashville, TN 37235, USA; Department of Electrical Engineering and Computer Science, Vanderbilt University, Nashville, TN 37235, USA; Vanderbilt University Institute of Imaging Science, Vanderbilt University, Nashville, TN 37232-2310, USA; Department of Radiology and Radiological Sciences, Vanderbilt University Medical Center, Nashville, TN 37232, USA; Department of Biomedical Engineering, Vanderbilt University, Nashville, TN 37235, USA

**Keywords:** face recognition, fMRI, higher order visual processing, parametric stimulus, white matter

## Abstract

Blood-oxygenation-level-dependent (BOLD) signals in magnetic resonance imaging indirectly reflect neural activity in cortex, but they are also detectable in white matter (WM). BOLD signals in WM exhibit strong correlations with those in gray matter (GM) in a resting state, but their interpretation and relationship to GM activity in a task are unclear. We performed a parametric visual object recognition task designed to modulate the BOLD signal response in GM regions engaged in higher order visual processing, and measured corresponding changes in specific WM tracts. Human faces embedded in different levels of random noise have previously been shown to produce graded changes in BOLD activation in for example, the fusiform gyrus, as well as in electrophysiological (N170) evoked potentials. The magnitudes of BOLD responses in both GM regions and selected WM tracts varied monotonically with the stimulus strength (noise level). In addition, the magnitudes and temporal profiles of signals in GM and WM regions involved in the task coupled strongly across different task parameters. These findings reveal the network of WM tracts engaged in object (face) recognition and confirm that WM BOLD signals may be directly affected by neural activity in GM regions to which they connect.

## Introduction

Functional magnetic resonance imaging (fMRI) has become a well-established technique to detect variations in neural activity in cortex by measuring blood-oxygen-level-dependent (BOLD) signals. These variations may arise as a result of a stimulus or task, or multiple voxels and/or regions of cortex may exhibit synchronized spontaneous fluctuations in a resting state, which are then identified as functionally connected within a brain network (Ogawa et al. 1992; Biswal et al. 1995; Fox and Raichle 2007). In both cases, in the vast majority of previous studies, BOLD signals in white matter (WM) have been consistently overlooked and, in practice, the average BOLD fluctuations in WM have often been considered as a nuisance covariate to be removed by regression in order to reduce physiological noise (Behzadi et al. 2007; Caballero-Gaudes and Reynolds 2017). In recent years, an increasing body of evidence indicates that BOLD signals within WM appear to reflect neural activities (Gore et al. 2019). For example, our previous studies have shown that WM voxels exhibit synchronized resting-state BOLD fluctuations within specific tracts (Ding et al. 2013, 2016; Schilling et al. 2019; Wu et al. 2019; Li et al. 2020). Further findings were reported by Marussich et al. (2017) and Peer et al. (2017), suggesting that segregated WM networks can be identified using a K-clustering method or independent components analysis based on the temporal patterns of signals in WM voxels, and these networks closely resemble known gray matter (GM) circuits and WM tracts. These works highlight the relevance of taking WM BOLD signals into account in resting-state studies because of their similar properties with those in GM. Along this line, Ding et al. (2018) demonstrated that resting-state BOLD signals in WM tracts are strongly correlated with signals in specific GM areas, and these correlations can be increased by specific functional demands. In addition to analyses that focus on spontaneous BOLD fluctuations during rest, Mazerolle et al. (2010) and Gawryluk et al. (2011) detected WM activation in the corpus callosum by performing the inter-hemispheric transfer task. However, the engaged areas reported are relatively small, possibly due to the lower vascular volume of WM as well as incorrect assumptions about the time courses of WM responses that are usually incorporated into for example, general linear models (GLM) for fMRI data analysis (Gawryluk et al. 2014). Li et al. (2019) comprehensively measured the hemodynamic response function (HRF) in WM using an event-related task and observed clear task-specific HRFs with significantly delayed onsets in WM tracts compared with activated gray matter, thus calling for modifications of standard methods of analysis of functional imaging data. To compensate for such temporal delays, Courtemanche et al. (2018) and Tae et al. (2014) used a set of HRFs with successive time shifts and observed that WM activity in the corpus callosum could be detected with increased sensitivity. These findings support the notion that neural activities are encoded in WM BOLD signals and are detectable using appropriate methods.

In previous studies exploring the visual system, we evaluated WM responses to simple sensory stimuli that activated primary visual areas and produced BOLD activation in both GM and WM relevant areas. For example, Huang et al. (2018) applied a periodic block-design visual task (8 Hz flickering checkerboard) and, instead of assuming any specific HRF for WM, conducted analyses based on the power spectra of the BOLD time series from each voxel. They observed that BOLD responses in both GM and WM voxels that were engaged in the task were also periodic with a strong component at the fundamental task frequency compared to other components. Mishra et al. extended this study by varying the frequency (2–14 Hz with a step-size of 2 Hz) of the flickering checkerboard so as to produce different intensities of visual responses. They observed concomitant variations in both cortical and associated WM regions (Mishra et al. 2020), and these variations were determined by a single task parameter. These findings suggest strongly that the magnitudes of WM BOLD signals reflect the degree of neural activity in associated GM regions. However, these studies have been limited mainly to low-level visual processes, and there remains a paucity of evidence on the neural coupling between WM and GM in response to higher-level visual tasks in which the responses are often weaker and which incorporate more complex cognitive processes. Here we aim to fill this gap in experimental studies of GM–WM correlations in a face recognition task.

There exists an extensive literature demonstrating that distinct brain regions exhibit highly reliable preferential responses to the recognition of human faces (Gauthier et al. 1999; Kanwisher and Yovel 2006; Doris Y. Tsao 2012). In particular, part of the fusiform gyrus responds strongly and selectively to human faces compared with other visual objects, and the presentation of faces elicits a characteristic N170 evoked potential that can be measured by electrodes at the surface of the head (Bentin et al. 1996). Horovitz et al. (2004) designed a parametric task derived from earlier work by Rossion et al. (2003) by adding different levels of noise into face pictures viewed during a scan and observed that the magnitudes of BOLD signals in the right fusiform face area varied as a function of the noise levels. A key finding from these studies is that responses in GM in occipitotemporal regions engaged in higher order visual processing can be modulated by an external task parameter, and this conclusion was confirmed by electroencephalographic (EEG) measurements performed in the same study. We adapted this paradigm to examine the GM–WM coupling under 6 different levels of face stimuli. First, a number of activated GM clusters were identified based on a localizer paradigm by contrasting trials showing faces vs. cars. Second, WM tracts were reconstructed using diffusion magnetic resonance imaging (MRI) data by first placing seeds at activated GM clusters. Finally, the BOLD time courses within these specific WM tracts were evaluated in terms of their magnitudes and temporal profiles across different levels of stimuli. Our data suggest that BOLD signals in both bilateral fusiform GM, and the WM to which they connect, exhibit a linear decrease in magnitudes with increasing noise in the face pictures. There are also significant correlations of BOLD magnitudes between activated GM areas and their connected WM tracts across different levels of stimuli. Moreover, BOLD fluctuations of selected WM tracts exhibit reduced magnitudes and delayed responses compared with the GM areas to which they connect. Our findings confirm that WM BOLD signals produced in response to complex visual recognition processes are coupled to those in connected GM and can be modulated by the same task parameters.

## Materials and Methods

### Subjects

This study was approved by the Vanderbilt University institutional review board. Written informed consent was obtained from each subject. Twenty healthy individuals (9 M/11 F; age, 31.7 ± 6.3 years) with no histories of neurological or psychiatric disorders were included.

### Task Design

First, a localizer task as shown in Supplementary Figure 1*a* (see supplementary data for details) was designed to identify GM areas responding preferentially to face presentations. In this task, 6 blocks of face pictures and six blocks of car pictures were presented alternately throughout the session (365 s). Each block lasted 20 s during which 20 pictures in succession were presented against a black background. Each picture was displayed for 700 ms and was followed by a blank interval of 300 ms. Blocks were spaced by 10-s of “resting-state” during which a gaze-fixation (a white crosshair) was displayed at the center of the screen. Following the localizer session, six runs of parametric face recognition tasks were acquired as shown in Supplementary Figure 1*b*. For each run, six blocks of face pictures were presented and spaced by 20-s resting-state blocks. Each block consisted of 20 face pictures to which was added a specified level of Gaussian noise (with zero mean). Six noise levels were determined in terms of the standard deviation of a Gaussian noise distribution (0, 0.025, 0.1, 0.5, 1, 5) as shown in Supplementary Figure 1*c*. The blocks of different noise levels were presented in a pseudorandom order over each run. The laser-scanned face pictures were provided by the Max Planck Institute for Biological Cybernetics in Tuebingen, Germany (Troje and Bülthoff 1996; Blanz and Vetter 1999), and the car pictures were obtained from the internet and were processed to remove backgrounds, and then histograms of voxel intensities were matched to the face pictures using a histogram equalization method (Garg and Jain 2017).

### MRI Acquisition

Images were acquired using a 3 T MRI scanner (Philips Healthcare, Best, Netherlands) installed at Vanderbilt University Institute of Imaging Science. Each subject was scanned in a supine, head-first position with restricting pads placed within the 32-channel head coil to ensure stability. BOLD-sensitive MRI images were acquired with repetition time (TR) = 2 s, echo time (TE) = 35 ms, SENSE factor = 2, matrix size = 80 × 80, field of view = 240 × 240 mm^2^, 34 slices of 4 mm thickness with a 0.5 mm gap. Pictures of faces or cars were visually projected onto a screen mounted in the back of the scanner and could be viewed by subjects through a mirror mounted on the head coil. To reconstruct WM tracts, diffusion-weighted MR images were acquired using a multishot, echo-planar imaging sequence with b = 1000 s per mm^2^, 32 diffusion-sensitizing directions, TR = 4.5 s, TE = 84 ms, matrix size = 112 × 112 × 68, and voxel size = 2 × 2 × 2 mm^3^. For both fMRI and diffusion MRI, 3 additional volumes were acquired with opposing phase encoding directions to estimate and correct for distortions. As anatomical references, high-resolution T1-weighted images were acquired using a 3-dimensional magnetization-prepared rapid gradient-echo sequence at a voxel size of 1 × 1 × 1 mm^3^.

### fMRI Analysis

Functional MRI images were preprocessed using the DPABI (Yan et al. 2016) and FSL (Jenkinson et al. 2012) and SPM (Friston 1994) software. First, the images were corrected for susceptibility-induced geometric distortions using images acquired with opposing phase encoding directions using the Topup tool in FSL. Second, the images were corrected for slice timing and head motion. Third, T1-weighted images were segmented into GM, WM, and CSF, and all these images were co-registered to the mean BOLD image resulting from the motion correction procedure. Fourth, the BOLD images were normalized into MNI space at a voxel size of 3 × 3 × 3 mm^3^, along with the segmented and co-registered T1-weighted images.

Following preprocessing, the signals measured for the localizer task (faces vs. cars) were convolved with a canonical HRF in the context of a GLM within SPM. On a single subject level, conditions were contrasted against each other to create a parametric image that reflected the signal changes with respect to faces compared with those of cars. On the group level, a one-sample *t*-test was applied to the parametric images across all subjects to create a map of the brain activation evoked by the faces for the whole population. Activated voxel clusters were reported at a threshold *P* < 0.05 (cluster level, two-sample *t*-test, family-wise error (FWE) rate corrected) with the cluster size larger than 25 voxels.

### Diffusion MRI Analysis

The raw diffusion-weighted images were first corrected for susceptibility-induced geometric distortions using images acquired with opposing phase encoding directions using the Topup tool in FSL. A 3 × 3 diffusion tensor was modeled for each pixel with multivariate linear fitting using DSI Studio software (http://dsi-studio.labsolver.org/). For each subject, the B0 image was co-registered to the T1 images and subsequently normalized to MNI space using SPM. This procedure was reciprocated and provided both forward (diffusion space toward MNI) and backward (MNI toward diffusion space) transformation matrices. To guide the reconstruction of tracts, the activated clusters of each subject were transformed from MNI to the diffusion space using the backward transformation matrix. We then performed diffusion-based tractography by considering these GM clusters as the seed regions to search throughout the rest of the brain for all possible voxels that connect to the seeds, which produced a tract density map in which the value of the density reflects the probability that WM fibers traverse the location. These maps were later transformed into the MNI space using the forward transformation matrices. A population-based density map was then computed by averaging across all 20 subjects. In MNI space, the population-based density maps were then thresholded at 30% of their maximal values to remove weak connections and masked with the WM mask at a threshold of 95% to exclude the influences from adjacent GM, as well as superficial WM voxels.

### Timecourse Analysis

The average time course within a task block was analyzed assuming a 40-second inter-onset interval (IOI), the time between the onsets of each successive block, as illustrated in Supplementary Figure 1*b*. The blocks with the same noise level were temporally aligned according to their onsets, and their time courses were averaged across runs and subjects. The magnitude of each timecourse was represented by the average magnitudes for time points in the range of [4–24 s] which approximated to the BOLD peak in response assuming the canonical HRF of the human brain (Lindquist et al. 2009). For WM we extended the time window to [4–30 s] to allow for the delayed response in WM (Li et al. 2019). The relationship between these magnitudes and noise levels was examined using Pearson’s correlation. A permutation test was used to examine the significance of the BOLD changes in these tracts in response to the task. For this, the magnitudes of *m* voxels that composed the tract were denoted by {*Vtr*} and the magnitudes of the remaining *n* voxels within the WM mask were denoted by {*Vwm*}. First, we calculated the difference of means between the 2 sets: *Df* = mean{*Vtr*} − mean(*Vwm*). Then the 2 sets were pooled to produce {*Vall*} = {*Vtr*; *Vwm*} which consists of *m + n* elements. We initiated the permuation by randomly sampling *m* elements from {*Vall*}, and calculated the difference in means between those *m* elements and the remaining *n* elements to produce *d1*. The permutation was repeated 5000 times and each time we obtained values of the difference *d1*, *d2*, *d3*, …, *d5000*. Finally, we generated the histogram of {*d*}, and the *P* value = (the number of permutations where *d* ≥ *Df*)/5000.

The GM–WM coupling was evaluated in terms of the Pearson’s correlation of magnitudes or time to peak (TTP) between activated GM clusters and the tract to which they connect across subjects and noise levels. To eliminate subject effects, the subject ID was regressed out from the variables before correlation. Note that the identifications of TTP were also limited within certain ranges: [4–24 s] for GM and [4–30 s] for WM. For comparison, Pearson’s correlation of magnitudes between GM clusters and 60 WM tracts were also assessed. Such tracts were defined in a WM atlas, namely, JHU-MNI-ICBM152, proposed by Oishi et al. (2008).

## Results

### Brain Activation in Response to Faces and Associated WM Tracts

**Figure 1:**
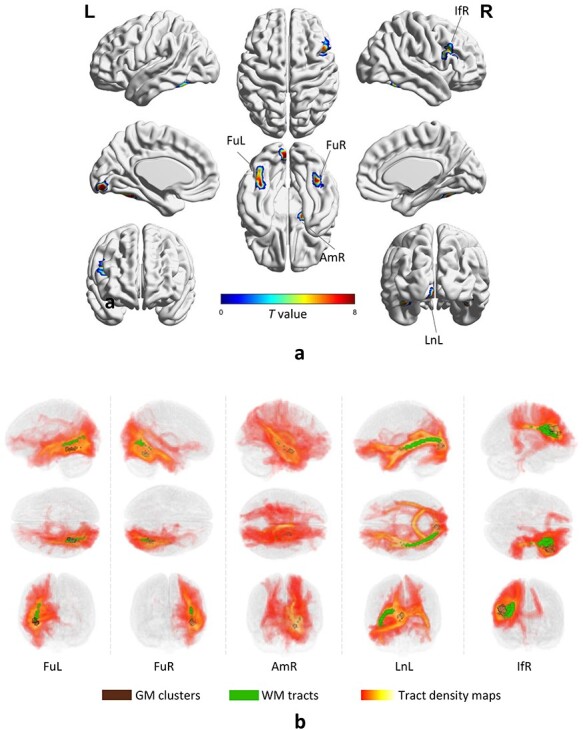
GM/WM areas that are involved in the task. (*a*) Five activated clusters (2-sample *t*-test, *P* < 0.05, FWE corrected) were distributed throughout the brain, including the bilateral fusiform gyrus (FuL and FuR), amygdala right (AmR), lingual gyrus left (LnL), and inferior frontal gyrus right (IfR). (*b*) The visualization of activated GM clusters (gray), tract density maps (red temperature) yielded by a tractography approach and placing seeds at activated GM clusters, and WM tracts (green) produced by setting a threshold to the tract density maps.

Five major activated GM clusters as shown in Figure 1*a* were reliably detected (*P* < 0.05, *t*-contrast, FWE corrected) across the population by contrasting faces to cars. Each cluster was identified by the name of an anatomical structure it overlaps to the maximum possible extent. These clusters include fusiform left (FuL, peak MNI coordinate [−39 –48 −21]), fusiform right (FuR, 42 −51 −21), amygdala right (AmR, [18 −6 −15]), Lingual gyrus left (LnL, [−6 −87 −9]) and a small area located at inferior frontal area right (IfR, [48 15 30]).

Seeds were placed at each of these GM clusters as shown in Figure 1*b* (gray areas), after which diffusion tractography was used in each subject to search for the possible paths that connect the seeds and the rest of the brain. This produced a density map, as shown in Figure 1*b* (red temperature scale), where the value of each voxel represents the probability the paths traverse this voxel. By setting a threshold to the population-averaged density map, a WM tract, as shown in Figure 1*b* (green areas), that represents the highest connection to each GM cluster was reconstructed. The result shows that the tracts connecting bilateral fusiform areas (FuL and FuR) expands toward the posterior area of the brain and briefly represents the temporal-occipital path. The tract connecting the AmR is small in size, expanding toward the parietal area of the brain. The tract connecting the LnL represents the core of the temporal-occipital path. For IfR, the tract was limited in an adjacent area of GM, possibly representing a U-fiber nearby.

### Time Courses and their Magnitudes in GM and WM across Different Task Parameters

**Figure 2:**
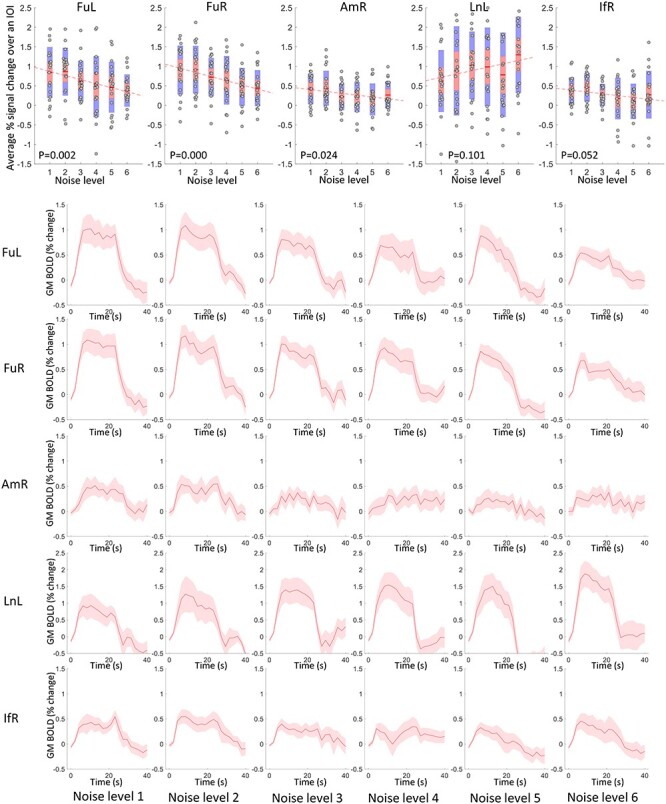
Time courses and their magnitudes of BOLD signals in activated GM clusters across 6 noise levels. Row 2–6: averaged time courses within 40-s IOI regarding five activated GM clusters in 6 noise levels. The centerline corresponds to the mean and the shade is the associated 95% CI. The *x*-axis indicates the time (s) and the *y*-axis indicates the magnitudes (% BOLD change). Row 1: The visualization of the average magnitude of the time course over the IOI for all subjects, plotted against the noise levels. *P* < 0.05 indicates a significant correlation.

**Figure 3:**
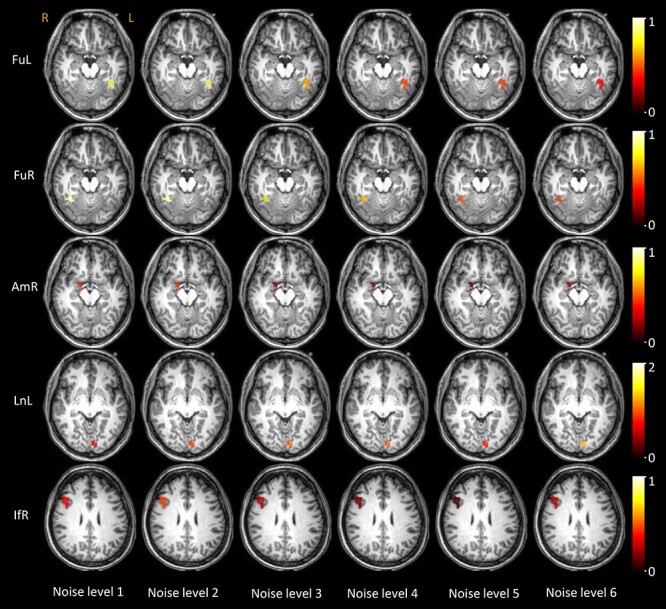
Visualization of the population mean of BOLD magnitudes on GM voxels that are analyzed across 6 levels of noise.

Figure 2 (row 2–6) shows the average time courses of different GM clusters in response to faces with varying levels of noise. FuL, FuR, AmR, and IfR exhibit decreasing magnitudes when adding more noise to the face pictures, whereas LnL exhibits increasing magnitudes. The average magnitude of the time course over the IOI is shown in a scatter plot as a function of noise level for each subject in Figure 2 (row 1), where the magnitudes of FuL, FuR, and AmR exhibit significant negative correlations with the noise level. In contrast, the average magnitude with respect to LnL shows a trend toward a positive correlation with the noise levels. The population mean of the magnitudes are superimposed on the GM voxels that were analyzed as shown in Figure 3.

**Figure 4:**
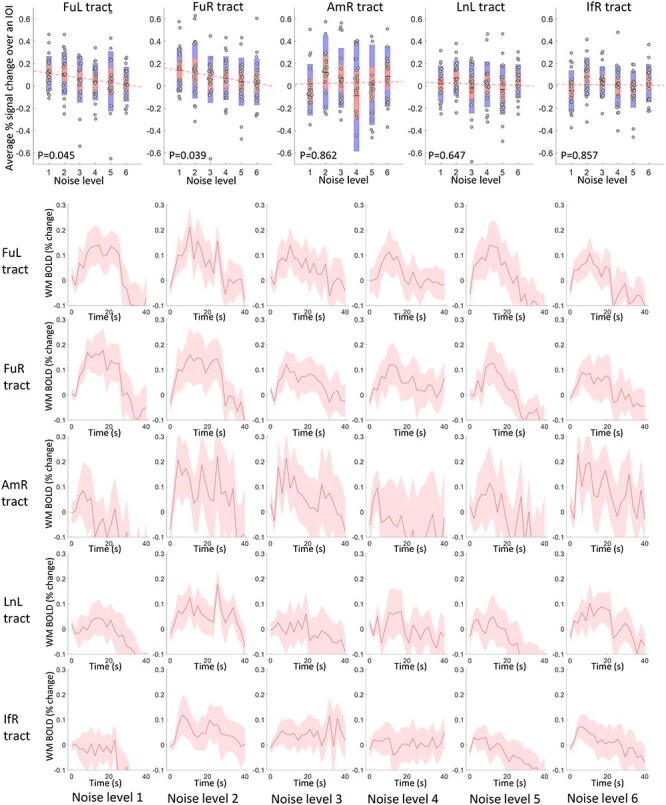
Time courses and their magnitudes of BOLD signals in the tracts that connect the activated GM clusters across 6 noise levels. Row 2–6: Averaged time within 40-s IOI regarding five tracts in 6 noise levels. The centerline corresponds to the mean and the shade is the associated 95% CI. The *x*-axis indicates the time (s) and the *y*-axis indicates the magnitudes (% BOLD change). Row 1: the visualization of the average magnitude of the time course over the IOI for all subjects, plotted against the noise levels. *P* < 0.05 indicates a significant correlation.

**Figure 5:**
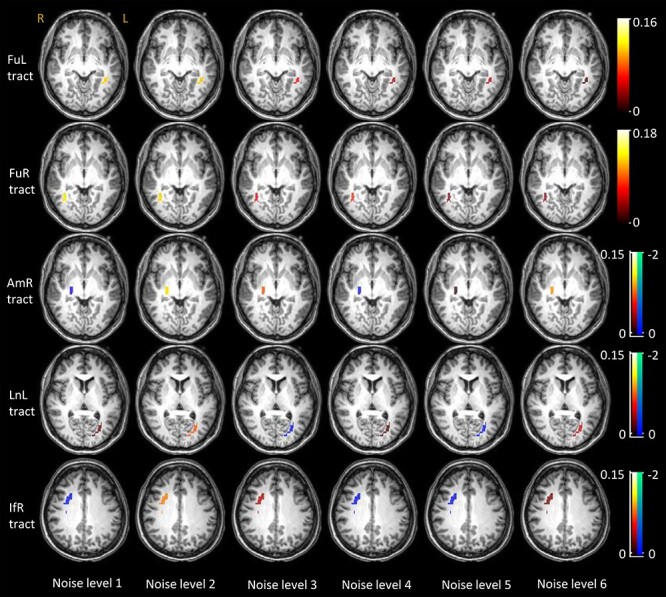
Visualization of the population mean of BOLD magnitudes on WM voxels that are analyzed across 6 levels of noise.

In a similar vein, the time courses of WM tracts that connect those activated GM clusters are shown in Figure 4 (rows 2–6), where the FuL and FuR tracts exhibit decreasing magnitudes as the noise level increased. The other tracts, particularly the AmR tract, showed higher variance in their time courses across subjects, and tended to show no significant changes with successive increases of the noise. This is further confirmed by the scatter plots of the average magnitudes over the IOI shown in Figure 4 (row 1), where the magnitudes of FuL and FuR tracts significantly correlate with noise levels. The population mean of the magnitudes are superimposed on the WM voxels that were analyzed as shown in Figure 5.

### Significance of the BOLD Change in WM Tracts in Response to Stimuli

**Figure 6:**
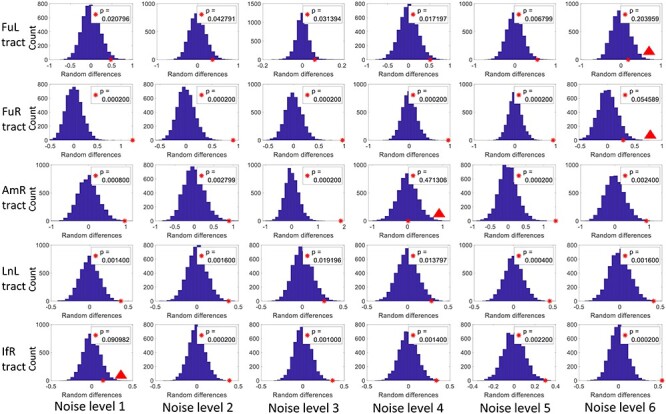
Histograms of differences (*d*) of the magnitude in means between randomly sampled WM and the rest WM voxels within a WM mask through 5000 permutations. The red asterisk indicates the observed difference (Df) in means between the voxels within the tract and the rest WM voxels. The *P* value was given by the potion of permutations where d ≥ Df (the portion of blue bins to the right side of the asterisk). *P* < 0.05 is considered a significant level. Non-significant BOLD changes are marked with red triangles.

A permutation test was used to examine the significance of the BOLD changes in the WM tracts in response to the stimuli. As shown in Figure 6, most of the tracts exhibit significantly higher (*P* < 0.05) magnitudes than the rest of WM voxels across all noise levels. The FuL and FuR show nonsignificant (marked by red triangles in Fig. 6) but a notable trend (*P* = 0.084 and 0.066) toward higher magnitudes in response to the noisest face pictures compared with the rest of WM voxels. A similar trend was also observed in the BOLD signals in IfR in response to the faces with level-1 noise. BOLD signals of AmR exhibit magnitudes that are equivalent to the rest of WM voxels (*P* = 0.469) at noise level 4. This observation is also supported by the time course shown in Figure 4, which shows large intersubject variability as well as magnitudes that are below the baseline. AmR showed significantly larger signals at other noise levels.

### GM–WM Coupling in Response to the Task with Varying Parameters

**Figure 7:**
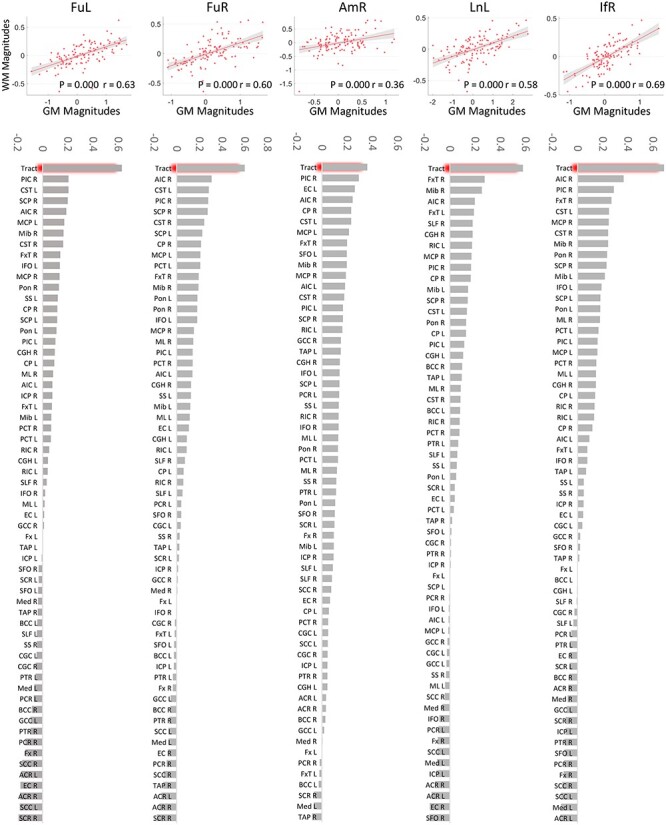
The GM–WM coupling in their magnitudes across all 6 levels of noise. Row 1: Correlation between magnitudes of BOLD signals of activated GM clusters and those of the WM tracts that they connect across subjects and noise levels. Note that subjects’ effects have been regressed out from the variables for correlation, and the *x* and *y*-axis represent the residuals rather than the real value of the magnitudes regarding GM and WM, respectively. Row 2: Comparing the correlation coefficients regarding structural connected GM–WM (the first bin) with those regarding structural irrelevant GM–WM pairs.

GM–WM couplings were analyzed by comparing the magnitudes of GM signals with those of WM across all 6 levels of noise. To eliminate subject effects, subject IDs were set as a covariate which was regressed out from the magnitudes of signals from GM clusters and WM tracts. In Figure 7 (row 1), each scatter plot indicates, for each subject, the BOLD magnitude (the residual after regression) of a GM cluster for a specific noise level versus that of its connected WM tract at the same noise level. This figure clearly shows that the signals in all GM clusters are significantly correlated with those in WM tracts to which they connect across different noise levels, supporting the notion that the responses in GM and corresponding WM are strongly coupled when the external stimulus changes. To further examine whether such coupling is specific to the structural relationship between GM and WM, we performed correlations between BOLD magnitudes of GM clusters and those of other WM tracts defined by an atlas. Figure 7 (row 2) shows that BOLD magnitudes in GM exhibit much higher correlations with those of the WM tracts to which they connect compared with other WM tracts. Four out of five bar charts indicate the largest correlation coefficients are much greater than the second-highest.

### Temporal Profiles of Coupled GM and WM

**Figure 8:**
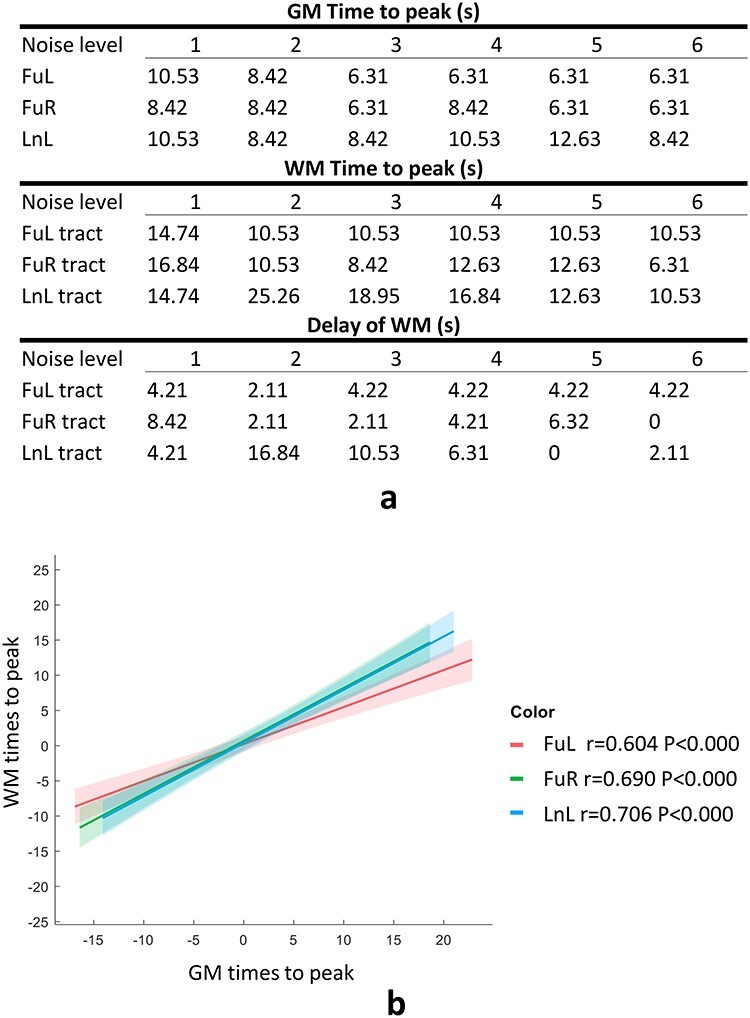
The GM–WM coupling in their TTPs across all 6 levels of noise. (*a*) Time to peaks of selected GM clusters and those of the WM tracts they connect. (*b*) Correlation between TTPs of BOLD signals of selected activated GM clusters and those of the WM tracts that they connect across subjects and noise levels. The centerline corresponds to the mean and the shade is the associated 95% CI. Note that subjects’ effects have been regressed out from the variables for correlation, and the *x* and *y*-axis represent the residuals rather than the real value of the times to peak regarding GM and WM, respectively.

We examined the TTP of the averaged BOLD responses for GM clusters and those of their connected tracts across noise levels. Three out of five tracts including FuL, FuR, and LnL exhibit a delayed peak in their averaged time courses compared with the GM clusters to which they connect at all noise levels, as shown in Figure 8*a*. We also observed that the maximal time peak was identified as 10.53 s for FuL and 8.42 s for FuR at noise level 1, whereas minimal TTP was identified as 6.31 for FuL/R at noise level 6. The times to peak of the WM tracts that connect FuL and FuR exhibit consistent patterns where maximal TTP was identified as 14.74 s for the left WM tract and 16.84 s for the right at noise level 1, whereas minimal TTP was identified as 10.53 s for the left WM tract and 6.31 s for the right at noise level 6. Correlation analysis of TTP between GM clusters and the WM tracts to which they connect was performed on a population basis across 6 noise levels, where subject IDs were regressed out to eliminate subject effects. Figure 8*b* indicates that the TTP of GM significantly correlated with that of WM with correlation coefficients of 0.604 for FuL, 0.690 for FuR, and 0.706 for LnL.

## Discussion

In this study, we evaluated GM–WM relationships in responses to a parametric face recognition task during which face pictures with 6 levels of added noise were viewed by the participants. We observed that bilateral fusiform areas, as well as the tracts to which they connect, exhibit clear, task-specific BOLD signal changes whose magnitudes vary as a function of the level of noise in the face pictures. The BOLD signal magnitudes in five GM areas that are involved in face recognition are significantly correlated with the signal magnitudes in the WM tracts to which they connect across different noise levels. Three out of these five WM tracts exhibit delayed responses compared with the GM to which they connect, and the times to peaks of their timecourses are strongly coupled to those of the GM to which they connect across noise levels.

In response to the localizer task (faces vs. cars), five GM clusters, including bilateral fusiform, amygdala right, lingual left, and an inferior frontal area right, were reliably activated. The fusiform face area was first described by Sergent et al. (1992) in a positron emission tomography (PET) study, and further evidence that it was specialized for the perception of faces was provided by various studies including EEG (Bentin et al. 1996; Rossion et al. 2003; Nguyen and Cunnington 2014) and fMRI (Clark et al. 1996; Wojciulik et al. 1998; Gauthier et al. 1999; Winston et al. 2004; Kanwisher and Yovel 2006). Whether this region is truly specialized for faces or rather reflects levels of expertise in identifying objects (Gauthier et al. 1999) does not affect the interpretation of our results. Compared with the fusiform, the rest of the activated clusters in this study presumably fulfill different roles in face recognition; for example, the amygdala and inferior frontal area are involved in processing emotional facial expressions (Jabbi and Keysers 2008; Wang et al. 2017; McFadyen et al. 2019), whereas the Lingual gyrus has been considered to be related to the processing of visual information about parts of human faces (McCarthy et al. 1999). Taken together, the activations we identified are involved in different cognitive processes engaged in face recognition and are in line with findings from previous studies.

Four out of five activated GM clusters (FuL, FuR, AmR, and IfR) exhibit decreasing magnitudes in their BOLD timecourses as the noise level in the face pictures increased. This finding is in agreement with previous work in which the BOLD signals in specific GM areas were modulated by the same parameters of the external stimuli during face recognition (Horovitz et al. 2004). It is reasonable to suppose that primary sensory areas will show a graded response to the effective magnitude of a stimulus, whereas other regions engaged in perception may exhibit a nonlinear (even binary) response. One unexpected finding is that the magnitudes of LnL responses exhibit an opposite trend with increasing noise, which could be a feature of the responses of lingual gyrus which, for example, is also associated with visual snow syndrome, a condition in which people see white or black dots in parts or the whole of their visual fields (Bou Ghannam and Pelak 2017). A fluorodeoxyglucose PET study has demonstrated hypermetabolism in the lingual gyrus in patients with visual snow syndrome (Schankin et al. 2020), where the “snow” is visibly consistent with the Gaussian noise we added into the face pictures. One of the most important findings of this study is that the two WM tracts that are connected with FuL and FuR exhibit decreasing magnitudes in their BOLD timecourses with increasing noise, suggesting that the BOLD responses of WM can also be modulated by a high-level visual processing task. In contrast, the magnitudes of the timecourses of AmR, LnL, and IfR exhibit nonmonotonic patterns across 6 levels of noises. However, BOLD signal magnitudes of all of these five tracts are significantly higher than those from random WM voxels, suggesting that WM responses are task-specific and highly dependent on the location of the tracts.

These findings may raise questions about whether WM BOLD signals reflect information processing intrinsic to the WM, whether they are passive reflections of communication between GM regions, or whether they are purely vascular couplings of no significance to neural function. By design, the WM responses detected are less likely to be attributable to potential confounding partial-volume effects with GM because the images were not smoothed and the WM analysis was limited by using a within subject-level WM mask at a tight threshold (>95%). Moreover, fMRI and diffusion images were corrected for distortions to eliminate possible contamination from adjacent GM voxels that may be misclassified as WM due to misregistration. Another common concern is that the WM signals are associated with physiological fluctuations, such as cardiac and respiration cycles. However, the magnitudes of such waveforms do not vary with the parameters of a task and thus are unlikely to contribute to the patterns we observed.

The magnitudes of the BOLD signals in selected tracts, whether or not they vary monotonically with the noise level, coupled well with those of GM clusters to which they connect. Potentially this coupling could arise from the residual effects of the oxygenation changes in GM vasculature draining into the WM. However, the WM receives blood almost entirely from medullary arteries which do not provide branches to GM (Nonaka et al. 2003) so it unlikely that blood flow out of an activated GM area can reach WM to modulate the signal there. We also observed that the correlation coefficients between interconnected GM and WM structures are much higher than those between pairs of GM and WM that are not anatomically connected. This suggests that such coupling is dependent on the structural relationship between GM and WM, supporting the observation of our previous study, based on an animal model and histology, which suggested that functional connectivity shares a similar pattern with the structural connectivity in the GM–WM network (Wu et al. 2019). Potentially, co-activations in response to specific functions in WM and GM may add a new dimension to our understanding of GM–WM connections using MRI, for example, WM tracts reconstructed on the basis of diffusion MRI. Particularly in studies of structure–function relations, it is often desirable to connect functionally activated GM with long-range WM tracts that transmit its functional signals, a mission that is made complicated by the presence of subcortical U-fibers at the GM–WM interface. Harnessing functional engagements of GM and WM may allow both structurally and functionally meaningful correspondences between GM and WM to be established. Similarly, crossing fibers within the WM itself may also be in principle disentangled by making use of different functional roles of individual fiber sub-populations. Although resolving fiber crossing based on functions necessitates high SNR and spatial resolution in fMRI, such a capability is becoming increasingly feasible by recent rapid advances in hardware and pulse sequences.

Consistent with our previous finding (Li et al. 2019; Mishra et al. 2020), we measured lower magnitudes and delayed responses in WM BOLD timecourses. This may be partly due to the lower vascular density and longer distances between feeding arterioles and areas of increased oxygen use in WM. We also observed a trend toward a decreasing TTP in FuL, FuR, and their corresponding tracts with increasing noise levels. Moreover, significant correlations of times to peak across noise levels were identified for GM clusters and the WM tracts they connect. This finding suggests that the temporal profiles, similar to the magnitudes, of the BOLD signals can be modulated by external tasks, and such profiles are coupled between GM and WM across different levels of stimuli. The current study uses a block-design task, which is powerful in terms of detection as the responses to a series of stimuli in a block could potentially increase the response amplitude. However, it does not allow the measurement of transient changes in brain activity, thereby providing little insights into the hemodynamic profile of neural response. Improved understanding of the coupling between GM and WM can be obtained by more accurate measurement of the temporal pattern of hemodynamic response and its underlying brain activity. Hopefully, this could be accomplished in our future work by leveraging event-related tasks, in which stimuli are short-lived and well-spaced, as well as high field imaging such as 7-T MRI, which could improve the resolutions along with the SNR of the images acquired.

Taken together, the evidence from this study confirms that WM BOLD signals may be evoked by a functional task and their magnitudes vary as a function of the parameters of the external stimuli. Furthermore, the magnitudes and the temporal profiles coupled well across different task parameters between GM and WM that are anatomically connected. These findings provide additional evidence for the existence of BOLD signals in WM related to neural activity and contribute to our understanding of how WM and GM are engaged in the performance of a cognitive visual task.

## Supplementary Material

Supplement_tgaa067
