# Supplementary material for: Identification of White Matter Networks Engaged in Object (Face) Recognition Showing Differential Responses to Modulated Stimulus Strength
Source: Cereb Cortex Commun. 2020 Sep 18;1(1):tgaa067. doi: 10.1093/texcom/tgaa067 (PMC7580301; doi:10.1093/texcom/tgaa067)
Supplement: Supplement_tgaa067 [file supplement_tgaa067.pdf]

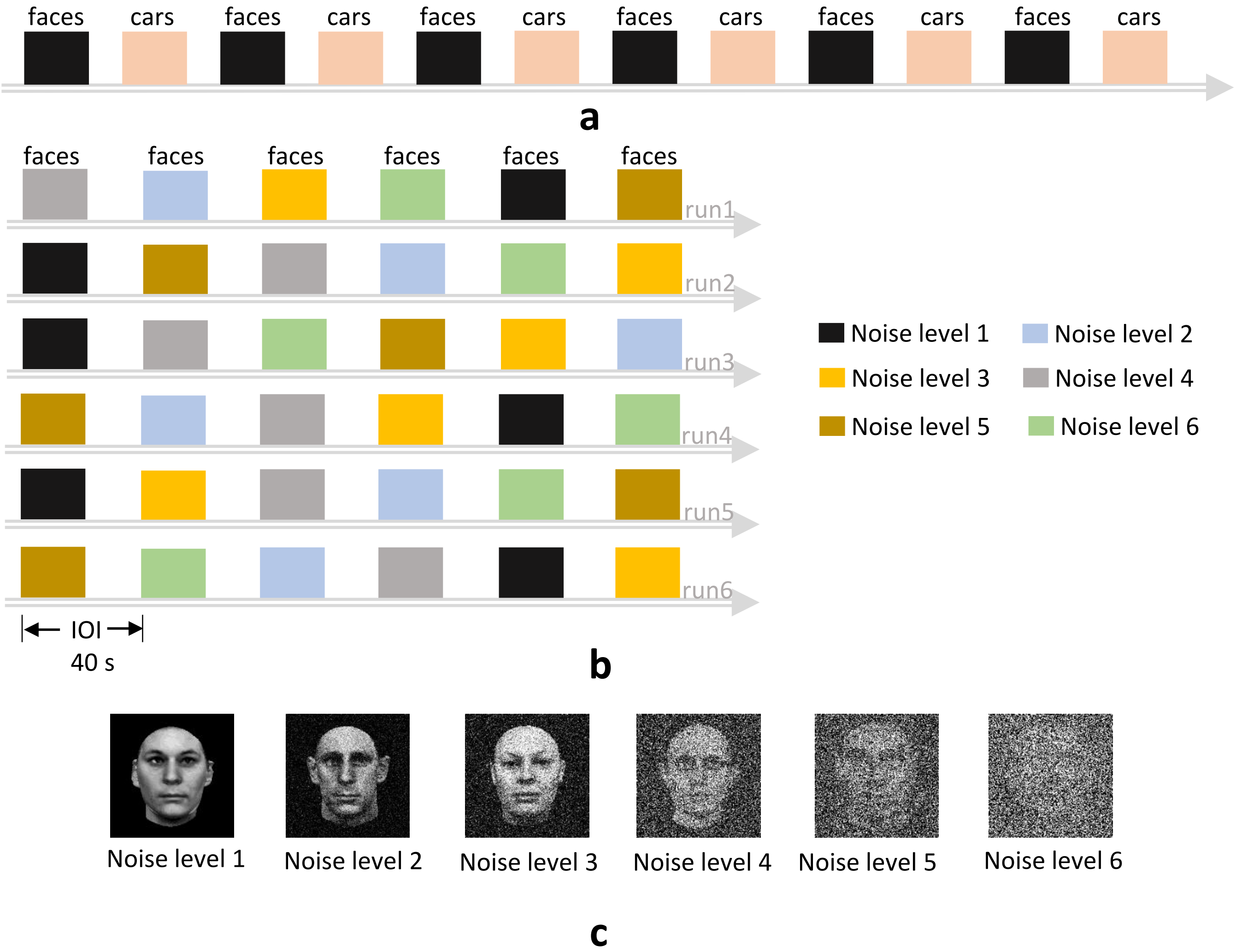

Figure S1. Experimental paradigms of the face recognition task. **a** Localizer paradigm used for identifying GM areas that respond to face recognition. Six blocks of face pictures and six blocks of car pictures were presented alternatively throughout the session. **b** Paradigm of six runs of parametric face recognition tasks. For each run, six blocks of face pictures with different levels of noise were presented in a pseudorandom order. **c** Examples of face pictures with six levels of noise.
